# Supplementary figures and images for: High-content synaptic phenotyping in human cellular models reveals a role for BET proteins in synapse assembly
Source: eLife. 2023 Apr 21;12:e80168. doi: 10.7554/eLife.80168 (PMC10121225; doi:10.7554/eLife.80168)

Source data - SYNAPSIN1 western blots

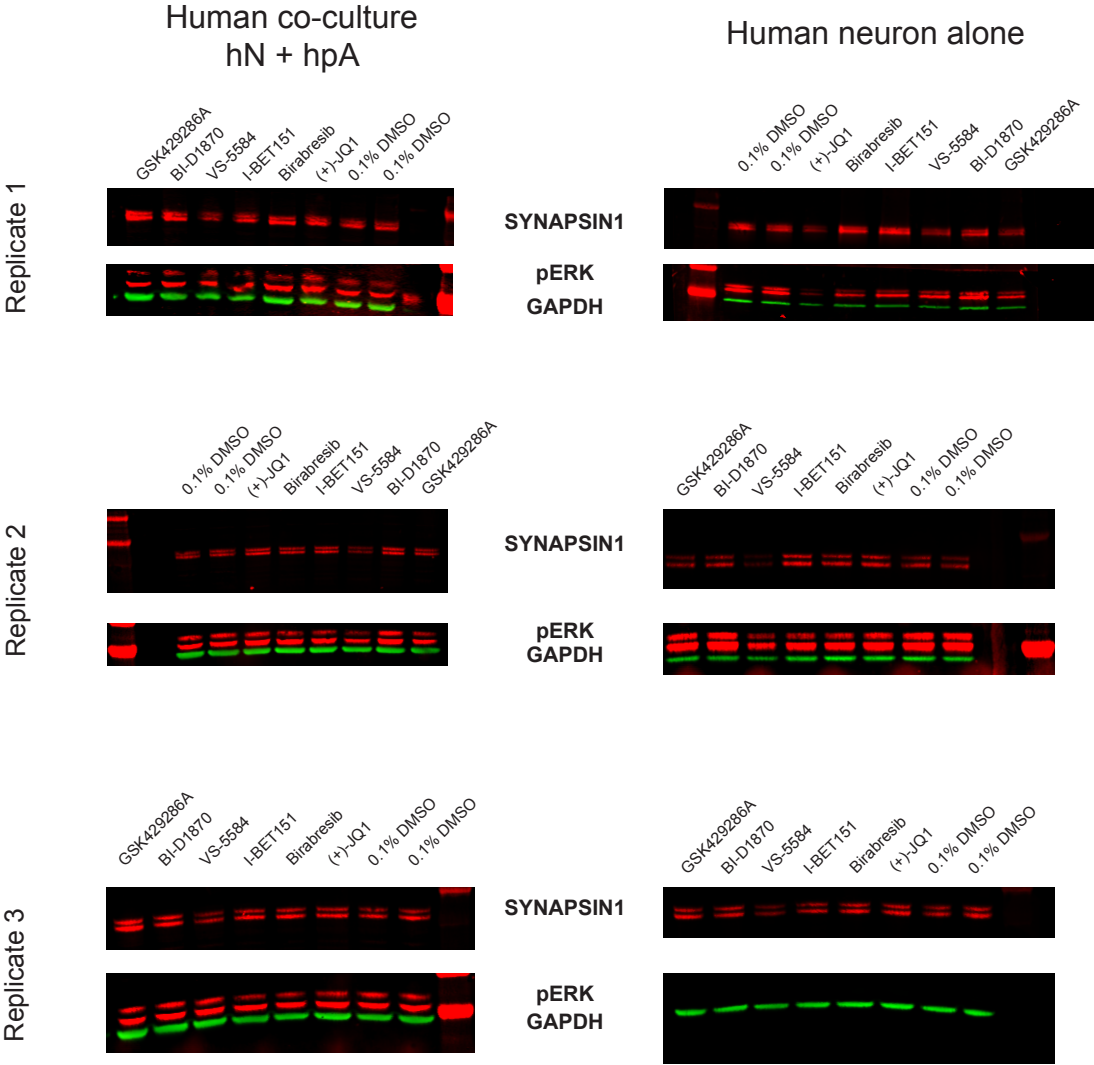

Supplement: Figure 4—source data 1. [file elife-80168-fig4-data1.zip › Figure 4-source data 1/Figure 4-source data.pdf]

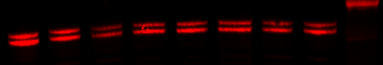

Supplement: Figure 4—source data 1. [file elife-80168-fig4-data1.zip › Figure 4-source data 1/co_WB_January19th2022_HmCC_Synapsin1.tif]

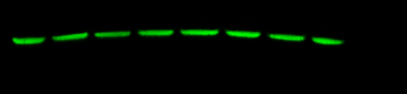

Supplement: Figure 4—source data 1. [file elife-80168-fig4-data1.zip › Figure 4-source data 1/mono_WB_January19th2022_HmNeurAlone_GAPDH.tif]

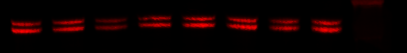

Supplement: Figure 4—source data 1. [file elife-80168-fig4-data1.zip › Figure 4-source data 1/mono_WB_January19th2022_HmNeurAlone_Synapsin1.tif]

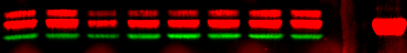

Supplement: Figure 4—source data 1. [file elife-80168-fig4-data1.zip › Figure 4-source data 1/mono_WB_January18th2022_pERKandGAPDH.tif]

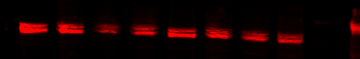

Supplement: Figure 4—source data 1. [file elife-80168-fig4-data1.zip › Figure 4-source data 1/co_WB_January11th2022_Synapsin1.tif]

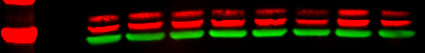

Supplement: Figure 4—source data 1. [file elife-80168-fig4-data1.zip › Figure 4-source data 1/co_WB_January13th2022_pERKandGAPDH.tif]

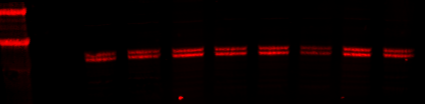

Supplement: Figure 4—source data 1. [file elife-80168-fig4-data1.zip › Figure 4-source data 1/co_WB_January13th2022_Synapsin1.tif]

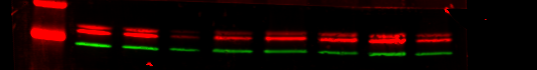

Supplement: Figure 4—source data 1. [file elife-80168-fig4-data1.zip › Figure 4-source data 1/mono_WB_January08th2022_ERK GAPDH.tif]

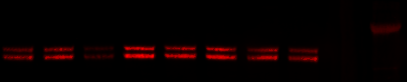

Supplement: Figure 4—source data 1. [file elife-80168-fig4-data1.zip › Figure 4-source data 1/mono_WB_January18th2022_Synapsin1.tif]

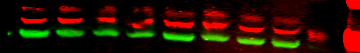

Supplement: Figure 4—source data 1. [file elife-80168-fig4-data1.zip › Figure 4-source data 1/co_WB_January11th2022_ERKandGAPDH.tif]

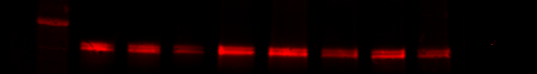

Supplement: Figure 4—source data 1. [file elife-80168-fig4-data1.zip › Figure 4-source data 1/mono_WB_January08th2022_Synapsin1.tif]

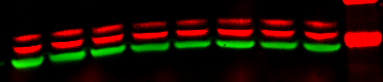

Supplement: Figure 4—source data 1. [file elife-80168-fig4-data1.zip › Figure 4-source data 1/co_WB_January19th2022_HmCC_pERKandGAPDH.tif]

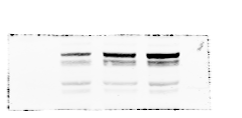

Supplement: Figure 6—source data 1. — Arrowheads indicate bands used for quantification. [file elife-80168-fig6-data1.zip › Figure 6-source data 1/BAIAP2 HmCC 7.19.21 DMSO JQ1 Bira auto exp.tif]

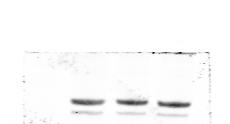

Supplement: Figure 6—source data 1. — Arrowheads indicate bands used for quantification. [file elife-80168-fig6-data1.zip › Figure 6-source data 1/Homer HmCC 1.17.21 HmCC ltr DMSo JQ1 Bira auto exp.tif]

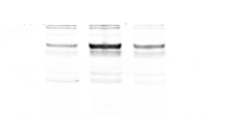

Supplement: Figure 6—source data 1. — Arrowheads indicate bands used for quantification. [file elife-80168-fig6-data1.zip › Figure 6-source data 1/BAIAP2 HmCC 3.31.21 ltr DMSO JQ1 Bira auto exp.tif]

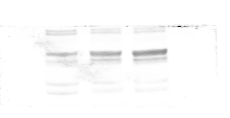

Supplement: Figure 6—source data 1. — Arrowheads indicate bands used for quantification. [file elife-80168-fig6-data1.zip › Figure 6-source data 1/BAIAP2 HmCC 1.17.21 HmCC ltr DMSo JQ1 Bira auto exp.tif]

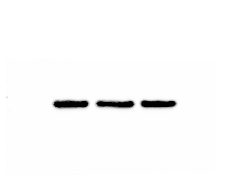

Supplement: Figure 6—source data 1. — Arrowheads indicate bands used for quantification. [file elife-80168-fig6-data1.zip › Figure 6-source data 1/CofHomer HmCC 7.19.21 DMSO JQ1 Bira high exp.tif]

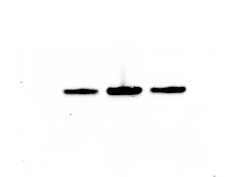

Supplement: Figure 6—source data 1. — Arrowheads indicate bands used for quantification. [file elife-80168-fig6-data1.zip › Figure 6-source data 1/Cof for Homer HmCC 3.31.21 ltr DMSO JQ1 Bira high exp.tif]

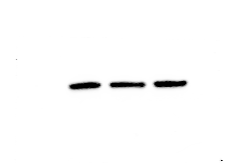

Supplement: Figure 6—source data 1. — Arrowheads indicate bands used for quantification. [file elife-80168-fig6-data1.zip › Figure 6-source data 1/CofBAIAP2 HmCC 7.19.21 DMSO JQ1 Bira high exp.tif]

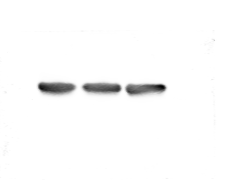

Supplement: Figure 6—source data 1. — Arrowheads indicate bands used for quantification. [file elife-80168-fig6-data1.zip › Figure 6-source data 1/CofBAIA HmCC 1.17.21 HmCC ltr DMSo JQ1 Bira auto exp.tif]

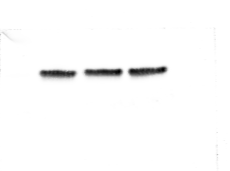

Supplement: Figure 6—source data 1. — Arrowheads indicate bands used for quantification. [file elife-80168-fig6-data1.zip › Figure 6-source data 1/Cofilin HmCC 7.9.21 ltr DMSO JQ1 Bira auto exp.tif]

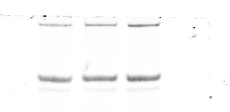

Supplement: Figure 6—source data 1. — Arrowheads indicate bands used for quantification. [file elife-80168-fig6-data1.zip › Figure 6-source data 1/Homer HmCC 7.9.21 ltr DMSO JQ1 Bira auto exp.tif]

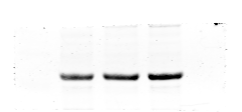

Supplement: Figure 6—source data 1. — Arrowheads indicate bands used for quantification. [file elife-80168-fig6-data1.zip › Figure 6-source data 1/Homer HmCC 7.19.21 DMSO JQ1 Bira auto exp.tif]

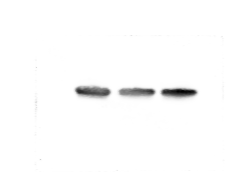

Supplement: Figure 6—source data 1. — Arrowheads indicate bands used for quantification. [file elife-80168-fig6-data1.zip › Figure 6-source data 1/CofiHomer HmCC 1.17.21 HmCC ltr DMSo JQ1 Bira auto exp.tif]

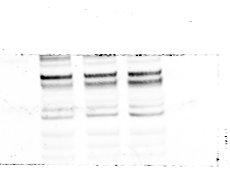

Supplement: Figure 6—source data 1. — Arrowheads indicate bands used for quantification. [file elife-80168-fig6-data1.zip › Figure 6-source data 1/BAIAP2 HmCC 7.9.21 ltr DMSO JQ1 Bira med exp.tif]

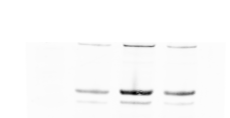

Supplement: Figure 6—source data 1. — Arrowheads indicate bands used for quantification. [file elife-80168-fig6-data1.zip › Figure 6-source data 1/Homer HmCC 3.31.21 ltr DMSO JQ1 Bira auto exp.tif]

Source data - BAIAP2 and Homer1 western blots

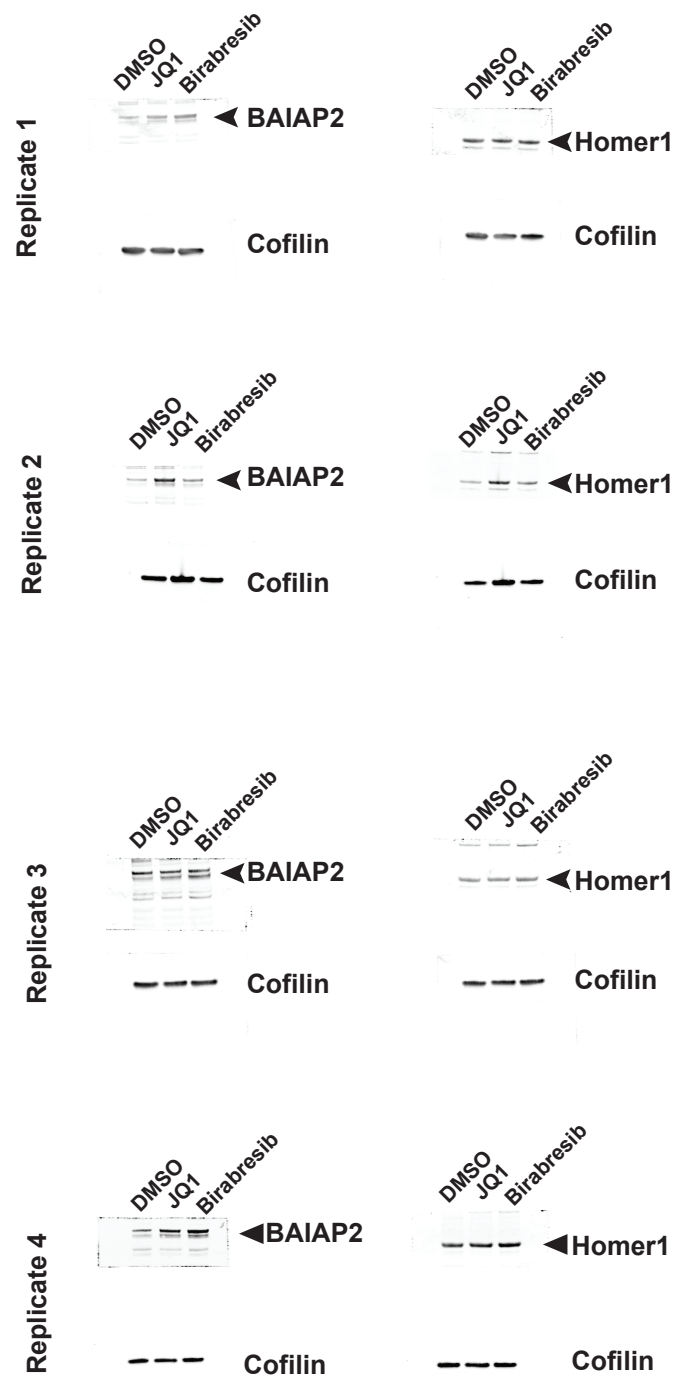

Supplement: Figure 6—source data 1. — Arrowheads indicate bands used for quantification. [file elife-80168-fig6-data1.zip › Figure 6-source data 1/Figure 6-source data.pdf]

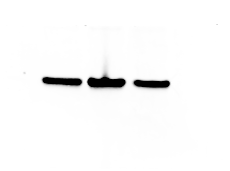

Supplement: Figure 6—source data 1. — Arrowheads indicate bands used for quantification. [file elife-80168-fig6-data1.zip › Figure 6-source data 1/cofilin HmCC 3.31.21 ltr DMSO JQ1 Bira high exp.tif]

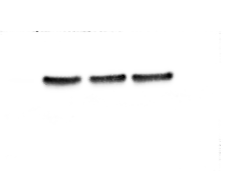

Supplement: Figure 6—source data 1. — Arrowheads indicate bands used for quantification. [file elife-80168-fig6-data1.zip › Figure 6-source data 1/Cof for BAIA HmCC 7.9.21 ltr DMSO JQ1 Bira auto exp.tif]
